# Endoscopic submucosal dissection with a traction-guided rendezvous approach for sigmoid colon cancer in the distal limb of a double-barrel colostomy

**DOI:** 10.1055/a-2777-4608

**Published:** 2026-02-05

**Authors:** Akimichi Hayashi, Hideyuki Chiba, Ai Hirohata, Toshifumi Iida, Yu Ebisawa, Jun Arimoto, Michiko Nakaoka

**Affiliations:** 1 74155 Department of Gastroenterology, Omori Red Cross Hospital, Tokyo, Japan

ESD is a minimally invasive treatment that allows en bloc resection of large tumors. However, it is sometimes difficult to obtain a full view and safely dissect under the tumor in a large lesion that occupies the lumen 1 2 . In this report, we present a case in which a giant protruded lesion occupying the lumen in the residual sigmoid colon after colostomy was successfully resected en bloc using a bilateral trans-stomal/anal approach with traction by a clip-with-line method (traction-guided rendezvous approach; Video 1 ).

A large type 0-Is colorectal tumor occupying the lumen in the residual sigmoid colon was resected using a traction-guided rendezvous approach.Video 1

**Fig. 1:**
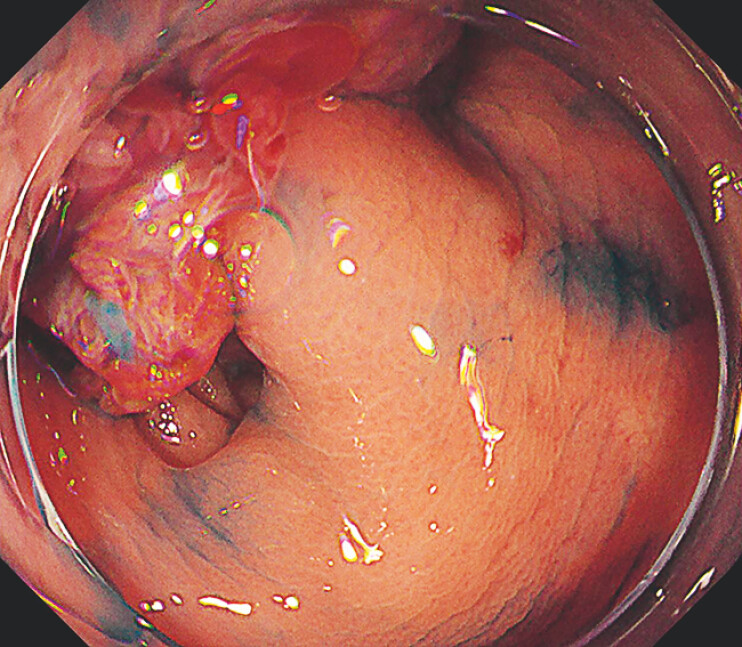
A large type 0-Is colorectal tumor occupying the lumen was identified in the residual sigmoid colon.

A 73-year-old woman had undergone a double-barrel transverse colostomy for perforated sigmoid diverticulitis 1 year earlier. The patient was referred to our department because of bloody stool from the anus, and a large type 0-Is colorectal tumor occupying the lumen was found in the residual sigmoid colon ( Fig. 1 ) and treated with ESD.

**Fig. 2:**
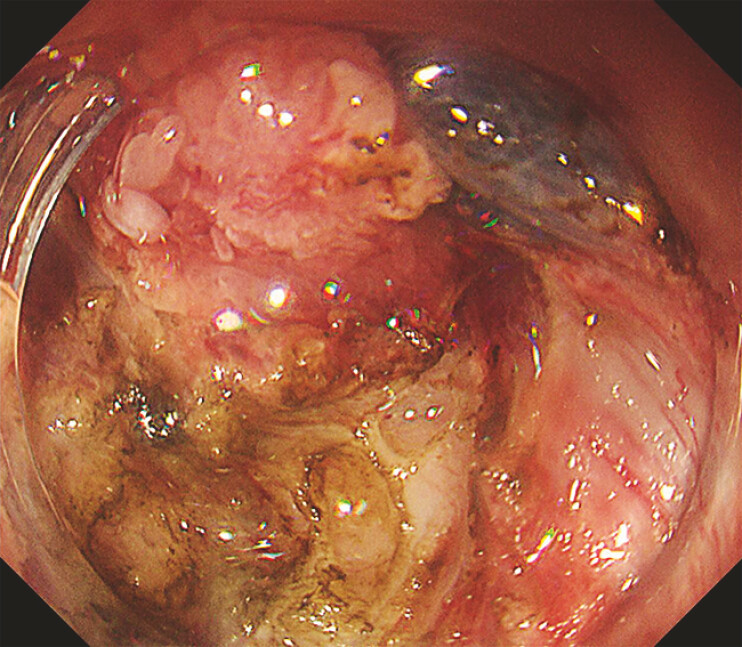
An endoscopic view after initial mucosal incision and flap creation.

**Fig. 3:**
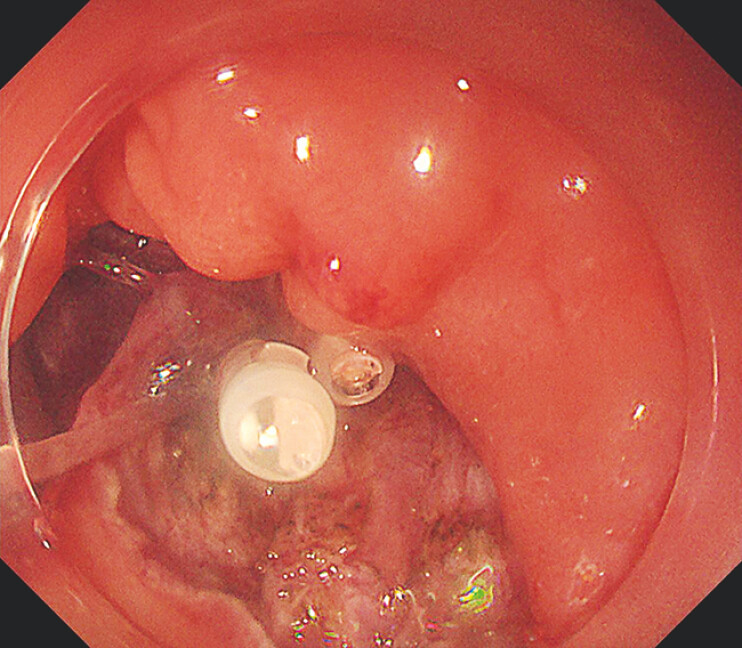
The base of the tumor was not clearly identified from the anorectal side due to the large bulge. Therefore, a clip-with-line method was placed from the stoma side.

**Fig. 4:**
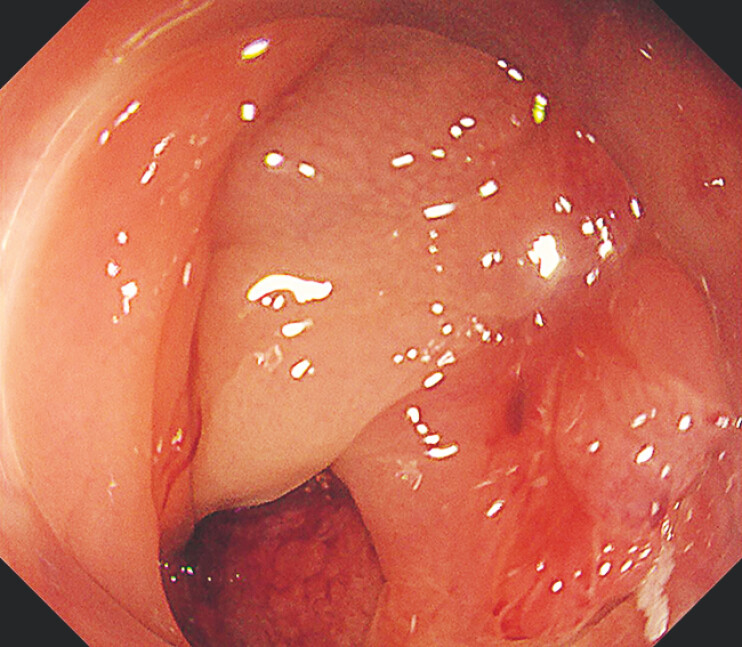
This traction using the clip-with-line method allowed us to identify the base of the tumor from the anorectal side.

**Fig. 5:**
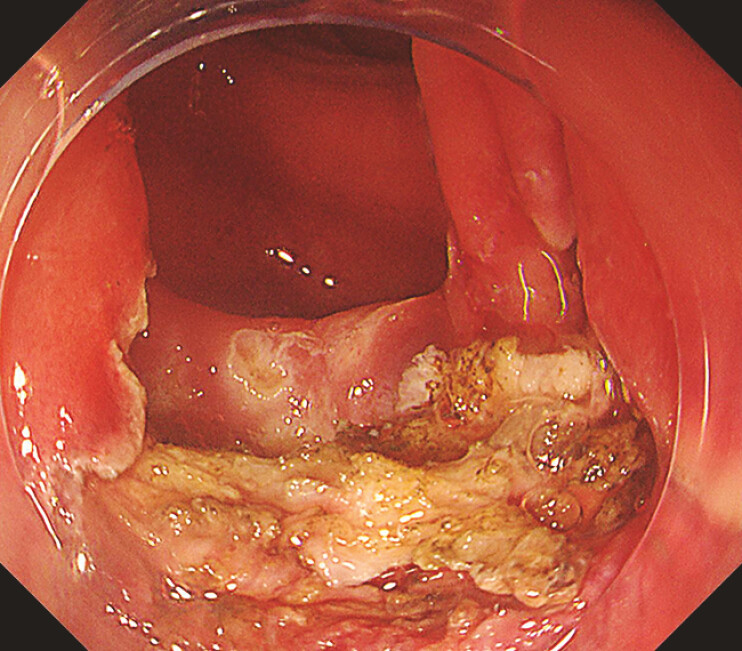
The muscle retracted sign and severe fibrosis were encountered, but the dissection was continued under traction, resulting in successful en bloc resection.

The initial approach was made from the stoma side, and a mucosal flap was created ( Fig. 2 ). The end point incision was made from the anorectal side. However, the base of the tumor was not clearly identified due to the large bulge. To overcome this limitation, a clip-with-line method was placed from the stoma side ( Fig. 3 ). This traction using the clip-with-line method allowed us to identify the base of the tumor from the anorectal side and the end point incision was successfully created ( Fig. 4 ). Re-access was made via the stoma side. Referring to the distal incision line, a complete circumferential incision was made. The muscular retracted sign and severe fibrosis were encountered, but the dissection was continued under traction, resulting in successful en bloc resection ( Fig. 5 ). The procedure was completed with endoscopic closure.

In this case, although the lesion was a giant protruded lesion occupying the lumen, the traction-guided rendezvous approach enabled safe and effective en bloc resection.

CL UCTN_Code_TTT_1AQ_2AD_3AD
